# Generative Artificial Intelligence in Primary Care: Qualitative Study of UK General Practitioners’ Views

**DOI:** 10.2196/74428

**Published:** 2025-08-06

**Authors:** Charlotte Blease, Carolina Garcia Sanchez, Cosima Locher, Brian McMillan, Jens Gaab, John Torous

**Affiliations:** 1 Participatory eHealth & Health data Research Group, Department of Women's and Children's Health, Uppsala University, MTC-huset, Dag Hammarskjölds väg 14B, 1 tr, Uppsala, 752 37, Sweden, 46 184710000; 2 Clinical Psychology and Psychosomatics, Faculty of Psychology, University of Basel, Basel, Switzerland; 3 Centre for Primary Care and Health Services Research, University of Manchester, Manchester, United Kingdom; 4 Department of Psychiatry, Beth Israel Deaconess Medical Center, Harvard Medical School, Boston, MA, United States

**Keywords:** generative AI, general practice, primary care, large language models, education, training, online survey questionnaire, qualitative research., artificial intelligence

## Abstract

**Background:**

The potential for generative artificial intelligence (GenAI) to assist with clinical tasks is the subject of ongoing debate within biomedical informatics and related fields.

**Objective:**

This study aimed to explore general practitioners’ (GPs’) opinions about GenAI on primary care.

**Methods:**

In January 2025, we conducted a web-based survey of 1005 UK GPs’ experiences and opinions of GenAI in clinical practice. This study involved a qualitative inductive descriptive analysis of a written response (“comments”) to an open-ended question in the survey. After analysis, the interpretation of themes was also informed by the technology acceptance model.

**Results:**

Out of 1005 respondents, 611 GPs (61%) provided written comments in response to the free text question, totaling 7990 words. Comments were classified into 3 major themes and 8 subthemes in relation to GenAI in clinical practice. The major themes were (1) unfamiliarity, (2) ambivalence and anxiety, and (3) role in clinical tasks. “Unfamiliarity” encompassed a lack of experience and knowledge, and the need for training on GenAI. “Ambivalence and anxiety” included mixed expectations among GPs in relation to these tools, beliefs about diminished human connection, and skepticism about AI accountability. Finally, commenting on the role of GenAI in clinical tasks, GPs believed it would help with documentation. However, respondents questioned AI’s clinical judgment and raised concerns about operational uncertainty concerning these tools. Female GPs were more likely to leave comments than male GPs, with 53% (324/611) of female GPs providing feedback compared to 41.1% (162/394) who did not. Chi-square tests confirmed this difference ((χ²₂= 14.6, *P*=.001). In addition, doctors who left comments were significantly more likely to have used GenAI in clinical practice compared with those who did not. Among all respondents, 71.7% (438/611) had not used GenAI. However, noncommenters were even less likely to have used it, with 80.7% (318/394) reporting no use. A chi-square test confirmed this difference (χ²₁=10.0, *P*=.002).

**Conclusions:**

This study provides timely insights into UK GPs’ perspectives on the role, impact, and limitations of GenAI in primary care. However, the study has limitations. The qualitative data analyzed originates from a self-selected subset of respondents who chose to provide free-text comments, and these participants were more likely to have used GenAI tools in clinical practice. However, the substantial number of comments offers valuable insights into the diverse views held by GPs regarding GenAI. Furthermore, the majority of our respondents reported limited experience and training with these tools; however, many GPs perceived potential benefits of GenAI and ambient AI for documentation. Notably, 2 years after the widespread introduction of GenAI, GPs’ persistent lack of understanding and training remains a critical concern. More extensive qualitative work would provide a more in-depth understanding of GPs’ views.

## Introduction

### Background

Since the launch of ChatGPT 3.5 (OpenAI) in November 2022, interest in large language model (LLM)–powered chatbots has burgeoned, with increasing attention given to their potential applications within clinical practice. These models, built on Generative Pre-trained Transformer architectures, undergo extensive pretraining on large datasets before fine-tuning for specific tasks. By leveraging probabilistic language generation, they produce contextually relevant responses and engage in dynamic, conversational interactions while retaining contextual memory, distinguishing them from traditional search engines.

The potential clinical utility of these models is now recognized. Emerging research highlights their capacity to streamline workflows by supporting automated medical documentation through “ambient” or “listening” artificial intelligence (AI) [1-4], enhancing the accessibility and empathy of patient-facing clinical notes [5,6], and assisting with differential diagnosis formulation [7-9]. However, significant challenges persist. LLM-powered tools are prone to factual inaccuracies, or “hallucinations,” and may perpetuate biases related to race, gender, and disability, contributing to algorithmic discrimination in health care [10,11]. In addition, the widespread availability of consumer tools, combined with their consummate capacity to engage users in “conversations,” raises significant concerns regarding patient privacy [12,13].

Despite growing enthusiasm, most studies have evaluated generative artificial intelligence (GenAI) in controlled environments rather than examining real-world clinical adoption. Limited research has explored how physicians, particularly those in frontline health care roles, integrate GenAI into daily practice. Addressing this gap, a 2023 survey of 138 psychiatrists affiliated with the American Psychiatric Association reported that 43% (59/138) had used ChatGPT-3.5, and 33% (45/138) had used GPT-4.0 (OpenAI) for clinical queries [14]. Similar findings emerged from a broader survey conducted in November 2023 among 938 UK public sector professionals, including National Health Service (NHS) staff members (225/938, 24%) and emergency service workers (141/938, 15%) [15]. In this cohort, 45% reported awareness of colleagues using GenAI, and 22% confirmed personal use. In February 2024, we conducted a nationwide survey of 1006 UK general practitioners (GPs) to assess their adoption of GenAI into clinical practice [16]. Among respondents, 20% (205/1006) had adopted GenAI tools, predominantly for documentation (47/160, 29%) and differential diagnosis support (45/160, 28%). Although interest in GenAI in health care is growing, empirical data on clinician adoption, particularly from surveys and qualitative studies, remain scarce.

### Objectives

Amid ongoing debates about AI’s impact on the future of medical professions, the perspectives of practicing physicians have received limited attention [14-16]. To address this gap, we conducted an online survey examining UK GPs’ experiences and opinions on the potential impact of GenAI in primary care. Recognizing the value of qualitative insights, we incorporated an open-ended survey question to explore GPs’ perspectives in greater depth. We aimed to provide a preliminary investigation into their views on how GenAI shapes primary care.

While the analysis followed an inductive, descriptive approach, we drew on the technology acceptance model (TAM) as a conceptual lens to help interpret the identified themes [17]. TAM is a widely used framework for understanding users’ adoption of new technologies. It posits that acceptance is primarily influenced by design features of technology and their perceived usefulness and perceived ease of use, which shape attitudes and behavioral intentions. In health care, TAM has been applied to explore clinicians’ responses to digital tools, thereby offering a helpful lens for interpreting GPs’ varied perceptions of GenAI. To illuminate these themes, we applied TAM to our analysis; its constructs can help clarify how GPs responded to GenAI, particularly considering their limited exposure and varying levels of enthusiasm or concern.

## Methods

### Main Survey

A complete methodological description of the survey is available elsewhere [18]. In summary, we conducted an online survey among random GPs registered with the Doctors website, the largest professional network for UK doctors affiliated with the General Medical Council (GMC). At the time of the study, the Doctors website had 254,741 members, representing approximately 65% of the 390,000 registered doctors in the United Kingdom. Eligible participants also included those who had opted in to receive survey invitations via email. The survey was part of a recurring monthly omnibus survey, which maintains a fixed sample size of 1000 participants. Respondents were required to complete all closed-ended questions to submit their responses; however, response to a single open-ended, free-text question was optional. Invitations to a random sample of GPs who are members of the Doctors website were distributed via email notifications or home page advertisements on the Doctors website, depending on user preferences. The survey was open from January 7, 2025, to January 26, 2025. During the period in which the survey was administered, 25,569 GPs were active on the platform. The online survey was divided into 4 sections, which explored participants’ experiences with, and opinions about, the use of GenAI in primary care. Before launch, the survey underwent pretesting and a pilot phase involving 5 UK-based GPs to assess usability and clarity. The final version of the survey (Multimedia Appendix 1) was designed to take 3‐5 minutes to complete. The study adhered to the CHERRIES (Checklist for Reporting Results of Internet E-Surveys) guidelines [1] (Checklist 1).

### Ethical Considerations

The study received ethical approval from the Faculty of Psychology, University of Basel, Switzerland (approval #030-24-1). Participants were assured of anonymity, and informed consent (Multimedia Appendix 2) was obtained before participation. The survey was hosted on the Doctors website’s secure platform, and all responses were encrypted and anonymized before data analysis. Email addresses and personal identifiers were removed before data transfer to the research team. The study complied with the European Union’s General Data Protection Regulation (GDPR). As an incentive, participants received a £7.50 (US $8.80, €8.83) shopping voucher upon survey completion.

### Qualitative Component

To maximize the response rate for the qualitative component, as noted, we included a single, optional, open-ended question that allowed participants to respond in more detail to the topic of the survey. Specifically, we requested: “Please add any comments about the topic or the survey. Please add 1‐2 brief comments.*”*

Descriptive qualitative data analysis was used, and we applied inductive thematic coding to the data [19,20]. Due to the limitations of the dataset—the brevity of comments and responses as sentence fragments—a full thematic analysis was not feasible [21]. Responses were cleaned (comments such as “none,” “n/a,” and “no comments” were deleted), and remaining responses were imported into QCAmap (coUnity Software Development GmbH) for analysis. To familiarize themselves with the data, CB and JT conducted multiple readings of the comment transcripts. Following this, an inductive coding approach was used, where descriptive labels (“codes”) were assigned to each comment. Comments containing multiple meanings were assigned multiple codes accordingly. To ensure consistency, comments and their corresponding codes were reviewed and compared to identify patterns, similarities, and differences. CB and JT collaboratively discussed coding decisions, leading to further refinements where necessary. Finally, despite the limitations with the dataset, first-order codes were organized into second-order categories based on shared meaning, providing a structured summary of the responses.

Although the coding process was conducted inductively, without the use of a predefined theoretical framework, we subsequently drew on constructs from the TAM to support interpretation of the findings, namely, perceived usefulness and perceived ease of use of GenAI [22]. This post hoc application of TAM helped us to consider how emergent themes might reflect underlying factors known to influence technology adoption, including perceived usefulness and ease of use.

## Results

### Overview

A total of 1141 unique visitors accessed the first page of the survey, and this number was used as the reference point for calculating view and participation rates. The view rate was considered 100% (1141/1141×100), reflecting direct exposure from both email and website recruitment channels. Of these, 1067 respondents provided consent to participate by selecting “yes” to the initial consent question, resulting in a participation rate of 94% (1067/1141×100). Among those who consented, 1005 respondents completed the full survey, yielding a completion rate of 94% (1005/1067 × 100). For clarity, 1141 represents the total number of unique visitors to the survey, 1067 the number of participants who consented, and 1005 the number who completed all survey items.

Out of 1005 respondents, 611 GPs (61%) provided written comments in response to the free-text question, totaling 7990 words. These comments were generally brief, ranging from short phrases to 1-3 sentences. As outlined in the quantitative survey, respondents were representative of UK GPs in terms of age and gender and from all regions of the United Kingdom [18]. GPs who submitted comments were not significantly different from those who did not, both in terms of role, age, practice size, or whether they think practice could be affected by GenAI (Table 1). GPs who submitted comments differed significantly from those who did not, particularly by sex. Female GPs were more likely to leave comments than male GPs, with 53% (324/611) of female GPs providing feedback compared with 41.1% (162/394) who did not. Chi-square tests confirmed this difference (*P*=.001). In addition, doctors who left comments were significantly more likely to have used GenAI in clinical practice compared with those who did not. Among all respondents, 71.7% (438/611) had not used GenAI. However, noncommenters were even less likely to have used it, with 80.7% (318/394) reporting no use. A chi-square test confirmed this difference (*χ²*=9.9, *P*=.002). More detailed information about these variables and their categories is available in Multimedia Appendix 3; deidentified, raw qualitative data are available in Multimedia Appendix 4.

**Table 1.** Demographic and practice characteristics of UK general practitioners who submitted free-text comments versus those who did not in a 2025 national survey on generative artificial intelligence in primary care.

Variable		Submitted comments (n=611), n (%)	Did not submit comments (n=394), n (%)	Comparison	
				Chi-square (*df*)	*P* value
Role: GP^a^ partner or principal		246 (40.3)	193 (49)	7.9 (3)	.05
Sex: female		324 (53)	162 (41.1)	14.6 (2)	.001
Age>46 y		334 (54.7)	212 (53.8)	0.04 (1)	.84
Practice place: large town or city (eg, Nottingham and Cardiff)		87 (14.2)	67 (17)	14.2 (5)	.01
Practice size>10,001		330 (54)	196 (49.7)	1.5 (1)	.21
Used GenAI^b^ to assist clinical practice: no		438 (71.7)	318 (80.7)	10.0 (1)	.002
Practice could be affected by GenAI: decrease my risk of having legal action taken against me		51 (8.3)	40 (10.2)	1 .04 (3)	.79

a GP: general practitioner.

b GenAI: artificial intelligence.

Through an iterative thematic analysis of the comments, three key themes were identified regarding GPs’ perspectives on GenAI in clinical practice: (1) unfamiliarity, (2) ambivalence and anxiety, and (3) role in clinical tasks. These themes were further divided into 8 subthemes, each detailed in the following sections with illustrative quotes (Textbox 1), where parenthetical numbers indicate individual participant identifiers. We emphasize that while Textbox 1 provides a summary of the major themes and subthemes, it is not intended as a hierarchical or conceptual model. Instead, it reflects a descriptive categorization based on the surface-level nature of the brief, diverse comments. For example, the subtheme “mixed expectations” captures a wide range of conflicting attitudes—from skepticism and fear to optimism—highlighting the spectrum of GP opinions rather than a single, discrete idea.

Textbox 1. **Summary of major themes and subthemes from UK general practitioners’ free-text comments about generative artificial intelligence. This textbox presents a descriptive classification; themes are not hierarchically ordered, and subtheme granularity varies, reflecting the range and specificity of the source data.**

**Unfamiliarity**

- Lack of experience and knowledge
- Need for training

**Ambivalence and anxiety**

- Mixed expectations
- Diminished human connection
- Skepticism about artificial intelligence accountability

**Role in clinical tasks**

- Help with documentation
- Questioning artificial intelligence’s clinical judgment
- Operational uncertainty

### Unfamiliarity

#### Lack of Experience and Knowledge

Many participants noted that they had “no experience” with GenAI or “haven’t used it.” Relatedly, multiple GPs stated that they had:

*Not enough experience of this area to comment*.

[#98, sex: prefer not to say, 46‐55 y, have not used]

This theme aligns with the broader survey finding that 75% of respondents had not used GenAI tools to assist with tasks in clinical practice [18]. However, some GPs noted limited familiarity with these tools; for example:

*Only started using recently…*

[#58, male, 46‐55 y, used]

*No experience in this at all but aware of it*.

[#132, female, 36‐45 y, have not used]

*Colleagues find it useful but I have not tried it yet*.

[#306, female, 36‐45 y, have not used]

Multiple respondents also identified their lack of knowledge about GenAI, and some GPs were emphatic about their lack of understanding; for example:

*The great unknown for me*.

[#242, female, 46‐55 y, have not used]

*Really no idea*.

[#823, female, 36‐45 y, have not used]

*Total minefield and I have no knowledge in this area*.

[#899, female, 36‐46 y, have not used]

#### Need for Training

Reported lack of training and guidance was another common concern. Some GPs stated that “GPs have no training in AI,” and expressed their “really limited exposure to AI in the workplace.” Many participants connected their lack of familiarity with GenAI with the need for greater formal guidance; for example:

*Need far more training on AI use in healthcare setting than currently available*.

[#997, male, 36‐45, No]

*I have little experience but I feel if we have some training […] this would be helpful in healthcare then I may be more comfortable with it*.

[#71, female, 36‐45 y, have not used]

*It needs to be delivered with inclusive training so less IT confident colleagues are not left behind*.

[#989, sex: prefer not to say, 36‐45 y, have not used]

A number of GPs emphasized that training needed to be specific to primary care; for example:

*Need more proactive support and resources to take on such tools to help support general practice utilize such tools*.

[#1056, male, 36‐46 y, have not used]

*I need more guidance on suitability for general practice in my location*.

[#259, male, 35 y or younger, have not used]

*…would be hesitant to use it without a significant amount to training tailored to a GP setting*.

[#772, female, 36‐45 y, have not used]

### Ambivalence and Anxiety

#### Mixed Expectations

Another major subtheme was the contrast in expectations surrounding GenAI. Many comments were brief, capturing broad sentiments about its potential impact. Notably, many GPs expressed deep skepticism or mistrust regarding AI’s role and consequences in primary care; for example,

*Not used it and frightened of it being implemented*.

[#1059, female, 36‐45 y, have not used]

*Theory sounds good. Dangerous in reality*.

[#515, male, 36‐45 y, used]

*I hope I get to retirement before all this nonsense takes over*.

[#637, male, 46‐55 y, have not used]

Some described GenAI as irrelevant to general practice; for example:

*To be honest AI not necessary in our domain*.

[#87, male, 46‐55 y, used]

*There is no role for AI in my role. We need real, not artificial, intelligence to look after patients*.

[#942, male, 46‐55 y, have not used]

Some GPs voiced anxiety, linking their negative expectations to “concerns about job security” and the existential threat of AI to their profession; for example:

*Could be the end of medicine as we currently know it*.

[#97, male, 46‐55 y, have not used]

*I dread to think of human doctors being replaced by AI*.

[#623, male, 46‐55 y, have not used]

*I would be very surprised if AI does not completely take over the role of GPs over the next 15 years*.

[#358, male, 46‐55 y, have not used]

*GPs are a dying breed. We had an informal discussion over our last Christmas meal if we need to now become IT professionals or even a bricklayer*.

[#985, male, 46‐55 y, used]

In contrast, many others expressed uncertainty and caution about the role of GenAI in clinical practice. For example:

*Unsure of its role yet*.

[#651, male, 56 y or older, have not used]

*I am not convinced that AI will help*.

[#113, female, 36‐46 y, have not used]

*Very early to say. Lots of unknowns*.

[#810, male, 36‐45 y, have not used]

However, reflecting the divergence of opinions, some GPs were “very excited about this development” and more optimistic about the usefulness of GenAI. For example:

*This is here to stay and if anything have increased presence in our working life. So we as clinicians need to embrace it!*

[#312, female, 36‐45 y, used]

*I can’t believe that it is taking so long to integrate AI into healthcare. It is very obvious that this is the way forward…*

[#42, female, 56 y or older, have not used]

*It will be transformational for healthcare*.

[#512, female, 46‐55 y, have not used]

*It’s a force for good*.

[#877, male, 46‐55 y, used]

#### Diminished Human Connection

Many GPs worried about the effects of GenAI on empathy and the risk that “it will take away the human touch,” undermine the face-to-face interaction, and that “it will dehumanize medicine.” For example:

*I feel AI will not read patients and miss the social cues and hidden agendas patients have*.

[#130, female, 36‐45 y, have not used]

Some respondents assumed that patients would view GenAI outputs as inferior when it comes to humanistic aspects of care; for example:

*Removes any element of compassion/empathy. No patient really wants to be seen by a robot. Would you?*

[#906, female, 35 y or younger, have not used]

*It may be a useful assistant but cannot replace the genuine interest, empathy and intuition of an experienced clinician*.

[#53, female, 36‐45 y, used]

*Not much empathy in the responses, sounds fake*.

[#569, female, 46‐55 y, have not used]

Some GPs, however, challenged this assumption and proposed that the adoption of GenAI tools might benefit communication and patient interactions; for example:

*A recent study showed that ChatGPT was more empathetic than doctors in dealing with patients […] I’m all up for technology*.

[#280, male, 46‐55 y, have not used]

*I think the gains from interpersonal interaction are being underestimated*.

[#479, male, 56 y or older, have not used]

*It’s good for patient communication*.

[#279, female, 36‐45 y, used]

#### Skepticism About AI Accountability

Comments reflecting GenAI anxieties also centered on the broader theme of AI access to sensitive patient information and accountability. However, again, many responses were brief and broadly framed. Some GPs specifically voiced concerns about patient data security, for example:

*Still concerns over third parties having access to patient data*.

[#475, male, 36‐45 y, have not used]

*Significant concerns regarding privacy and data protection*.

[#435, male, 46‐55 y, have not used]

*We use very little due to GDPR concerns, regulatory concerns, and do not use it in direct patient care currently…*

[#841, female, 36‐45 y, used]

Other GPs expressed attenuated optimism, describing the need for specific safeguards before AI could be adopted; for example:

*I’d like to use AI for note taking during consultations but difficult to implement in terms of privacy, confidentiality, security*.

[#211, male, 36‐45 y, used]

*Seems like a really positive way forward. Need to be sure about ethics re info sharing*.

[#686, female, 46‐55 y, used]

Other GPs raised concerns about legalities, particularly in relation to who, or what, would be accountable if things went wrong in the adoption of these tools; for example:

*I have concerns about who will regulate the provision of AI tools, and what impact their use will have on liability for medical errors*.

[#594, male, 56 y or older, have not used]

*Very suspicious of AI, it depends totally on quality of information inputted. Who is medicolegally responsible?*

[#629, male, 46‐55 y, have not used]

### Role in Clinical Tasks

#### Helps With Documentation

A dominant subtheme, when it came to the role of GenAI, was in administration and clinical documentation. These comments elaborate on quantitative findings indicating that documentation was the most common use case among GenAI adopters [18]. Drawing on TAM, these comments reflect a high level of perceived usefulness for GenAI tools in these administrative tasks, particularly where GPs cited time-saving benefits, improved workflow, and reduced cognitive burden. Many GPs expressed buoyancy about the capacity for these tools to “help efficiency” in clinical notes; for example:

*Completely changed my enjoyment of job – means I can focus on pt [patient] rather than busily typing away*.

[#311, female, 36‐45 y, used]

*Useful to speed up documentation*.

[#54, female, 36‐45 y, used]

Notably, many GPs specifically mentioned “Heidi” as an ambient clinical AI integrated into NHS trusts, which automates medical scribing, documentation, and patient data management with the aim of reducing administrative burdens for GPs. Respondents explicitly mentioned their positive experiences with this tool;

*Heidi Health has been incredible for generating excellent documentation for consultations that improve communication and reduce my workload as GP. Very accurate with medical jargon used*.

[#1017, male, 36‐45 y, used]

*Using Heidi as an ambient AI has made a massive improvement on my working efficiency and quality of notes*.

[#252, male, 36‐45 y, used]

*Heidi is fabulous. It saves me an hour a day but also a lot of brain ache*...

[#819, male, 46‐55 y, used]

However, some GPs expressed more reserved views about the role of AI in documentation, acknowledging the benefits while maintaining a cautious perspective:

*Saves time transcribing consultation and generating referral letters. Not perfect but helps*.

[#1043, female, 36‐45 y, used]

*Find it good for note taking and I guess about 90% accurate*.

[#310, male, 46‐55 y, used]

*I find that using Heidi intermittently will significantly reduce my documentation time. You do need to read the transcription prior to use in the notes […] but otherwise I find it very useful especially with more complex histories or mental health patients who may take longer consulting times*.

[#160, female, 36‐45 y, used]

Finally, a number of GPs specifically mentioned the role of GenAI in drafting patient-facing letters and improving communication, including for bridging language barriers; for example:

*Good for doing letters and complaint responses*.

[#758, female, 36‐45 y, have not used]

*Have found it helpful to re-phrase content sent to patients to make it easier to understand or remove jargon*.

[#967, male, 46‐55 y, used]

*It helped me with communication and good language with the patients as English is not my first language*.

[#57, female, 35 y or younger, have not used]

#### Questioning AI’s Clinical Judgment

Despite GPs’ optimism about the role of GenAI in documentation, most GPs were cynical or expressed reservations about the role of these tools in replacing doctors’ clinical judgment. Notably, however, it was unclear if these judgments encompassed user experiences; for example:

*AI cannot replace the spidey sense of a GP to know that something is wrong*.

[#32, male, 36‐45 y, used]

*It has the potential to aid in management of patients but there is a long way to go for its diagnostic accuracy*.

[#1034, female, 36‐45 y, have not used]

*There are times in my profession I used my “GUT” and it has been pretty good to me so far. People do not fit into boxes so I think AI will work on probabilities*.

[#933, male, 36‐45 y, have not used]

#### Operational Uncertainty

While less frequently mentioned, some participants stressed that AI must be “very carefully implemented” and require “clinical oversight,” highlighting the need for clearer divisions of labor between AI and doctors. One GP highlighted the following concern relating to doctors’ overreliance on AI:

*I use ChatGPT frequently and it has enormous potential and is very useful but it needs sense checking and it could cause complacency in busy overstretched healthcare professionals. When you start using it, it appears to have a solution to many problems and it also converses with you in a way that feels authentically human. These are impressive qualities but it should only be seen and used to augment rather than replace effort*.

[#922, male, 46‐55 y, used]

Taking a different angle on operational uncertainties, a minority voiced concerns about technopolitical shifts and the broader trajectory of AI’s impact. For example:

*Too many billionaires controlling our work as it is. Stuff AI doing turning us into robots*.

[#324, male, 46‐55 y, have not used]

*I believe AI is a Pandora’s box that has already been opened. We lack the safeguards or infrastructure to control its possible (and potentially catastrophic) effects*.

[#209, male, 46‐55 y, have not used]

## Discussion

### Principal Findings

This qualitative study offers timely insights into how GPs in the United Kingdom perceive the role of GenAI in primary care. While the analysis captures broad patterns and recurring sentiments, it is based on brief, free-text responses and should be understood as a high-level exploration rather than an in-depth account of underlying motivations or complex experiences.

Comments were classified into three major themes in relation to GenAI: (1) unfamiliarity, (2) ambivalence and anxiety, and (3) role in clinical tasks. Many GPs reported a lack of experience and familiarity with GenAI, and a need for training tailored to primary care. They also expressed a spectrum of views on GenAI in clinical practice, ranging from optimism to deep skepticism. While some saw GenAI as a promising tool for reducing administrative burden (particularly in documentation), others voiced concerns about its accuracy, potential to erode human connection, and unclear medico-legal implications. Given that GenAI users were more likely to provide comments, it is possible that certain benefits, particularly those related to documentation and workflow, may be somewhat overrepresented in the thematic analysis. In addition, widespread uncertainty about AI’s role in primary care, alongside ethical and regulatory concerns, underscored GPs’ reported need for structured guidance. This uncertainty, particularly around usefulness and ease of use, aligns with constructs from TAM [17], which posits that these perceptions are central to whether users adopt or reject new technologies.

This qualitative study supports other research indicating that GPs have limited formal training in AI, which directly influences their perceived ease of use—a central construct of TAM. The comments reflecting confusion, lack of confidence, or the need for guidance reveal how uncertainty about how to interact with GenAI tools may reduce behavioral intention to adopt them [15,23-26]. For example, a recent survey of 175 UK medical students found little awareness of formal AI or data science training in their curriculum, while 92% of 210 trainee doctors felt their AI education was inadequate. In our study, lack of familiarity with these tools was clear, with many GPs reporting minimal exposure to GenAI, stating they had no experience or understanding of how these technologies function; furthermore, this unfamiliarity was closely linked to GPs’ concerns about insufficient training. Online training is now available via the NHS Learning Hub. Certification as an “Artificial Intelligence Practitioner” is offered [27], and the Medical Schools Council has called for AI training integration and proposed learning competencies [28]. Our findings emphasize the critical need for AI-related training, which is both essential and highly sought after by GPs. From a TAM perspective, improving training could enhance both perceived ease of use and perceived usefulness (2 key factors) in shaping future adoption of GenAI tools in clinical practice [17,29]. Our findings also highlight the importance of developing innovative, adaptive training approaches. Given the rapid evolution of AI, GPs may be hesitant to invest time in education that risks becoming quickly outdated. Our results suggest that GPs seek training that clarifies the parameters of AI use, provides insights into its strengths and limitations, and offers accessible advice on staying updated as the technology advances.

GPs in our study expressed highly divergent views on GenAI in primary care, with opinions ranging from deep skepticism and existential anxiety about their job to excitement. These findings echo previous research, which shows physicians have mixed opinions about AI [23]; again, this broad spectrum of expectations may be indicative of a lack of formal education. These ambivalent or anxious attitudes, ranging from existential fears to cautious optimism, map onto TAM’s concept of attitudes toward technology, which in turn shape behavioral intentions. Where GPs felt hopeful, behavioral intention appeared stronger; where they expressed anxiety or confusion, adoption seemed less likely [29]. In addition, GPs’ varied interpretations of what GenAI entails may have shaped the divergence in views, contributing to both inflated concerns and enthusiastic projections.

Similar to previous research, GPs worried about the potential for AI to erode human connection and to undermine empathy, depersonalizing care [23,30]; this perspective is also common among leading medical commentators [31]. However, these views contrast with emerging research suggesting that GenAI may enhance, rather than diminish, compassionate care. In a blinded study, ChatGPT’s responses were rated as 10 times more empathetic than those of physicians, challenging assumptions about AI’s limitations in patient communication [6]. Other research suggests that AI can play a role in assisting clinicians to write empathetic communication with patients [32] and that patients cannot discern when AI is responding [33]. While more research is needed to explore patients’ opinions about using AI in clinical care, emerging studies suggest that at least some patients are turning to these tools for support [34]. Few participants commented on how AI and GPs might collaborate more effectively. The concept of AI as a “co-pilot” is an increasingly proposed model, drawing parallels to how airline pilots work alongside autopilot systems in aviation [35]. Others dispute the seamless integration of AI and doctors [36,37], challenging this assumption.

Many expressed concerns about AI’s accountability and data privacy. These are ongoing concerns. Implemented in February 2025, the European Union’s AI Act, the most comprehensive global legislation on AI, designates consumer GenAI tools as “high risk” due to concerns over cybersecurity, accuracy, reliability, and transparency [38]. Most LLMs do not claim clinical suitability, and the GMC advises physicians to apply professional judgment when using AI or emerging technologies in practice [28]. Our findings suggest that this guidance is insufficient to alleviate GPs’ concerns about accountability, privacy, and when it is appropriate to use these tools. Without a firm legal grounding, GPs may take on liability for AI errors and so place themselves at risk.

Notably, however, GPs were buoyant about the role for GenAI chatbots and ambient AI, particularly the medical AI scribe “Heidi Health,” in assisting with documentation. This finding aligns with previous surveys suggesting that doctors perceive AI as beneficial in handling administrative tasks [23,24,39,40]. Emerging studies also suggest AI may improve workflow efficiency [3,4]; some preliminary studies suggest it might reduce burnout [41,42]. However, the risk of GenAI hallucinations and errors raises concerns about the need for clinical oversight. These concerns may temper perceived usefulness—an essential driver of adoption according to TAM [17,29]—and reinforce GPs’ hesitation to rely on GenAI tools for clinical decision-making.

In our study, GPs questioned whether AI could ever replace the intuitive and experiential judgment of an experienced GP, emphasizing the irreplaceable nature of human clinical reasoning. While AI tools are not intended to replace clinical judgment, emerging evidence underscores their remarkable capabilities in differential diagnosis, with several studies demonstrating that GenAI can outperform physicians in certain diagnostic tasks [8,43]. However, many GPs may be correct in remaining unsure of the added value of AI-driven diagnostic support, especially if actual clinical care is based on real-world data and decisions are made on complex information instead of simple vignettes.

Notably, few GPs highlighted how GenAI tools might enhance patient access to medical information, suggesting a limited awareness of their potential role in patient empowerment. This limited awareness may reflect early-stage exposure, where both perceived usefulness and ease of use remain unclear, further influencing low behavioral intention to integrate such tools (again, consistent with TAM) [17,29]. In addition, despite well-documented concerns about algorithmic fairness and reliability, there was scarce mention of the risks of bias inherent in these AI-driven systems [10]. This lack of engagement with both the opportunities and risks of GenAI suggests a broader gap in discourse and critical evaluation of AI’s value in primary care, underscoring the need for more comprehensive discussions on its integration and ethical implications.

### Strengths and Limitations

This study represents the largest investigation to date into the perspectives of physicians on the integration of GenAI in clinical practice. Using a web-based survey may have encouraged more candid feedback, as evidenced by the strength of participants’ responses. As previously noted [18], although our sample aligned with the UK GP population in terms of age and regional distribution, as reflected in the GMC Registry, our sample was not gender-representative [44]. While the national GP workforce comprises more women than men (58% vs 42%), our sample had an equal gender split.

However, the study has limitations related to the use of a nonprobability sample via the Doctors website. We also acknowledge that the qualitative data analyzed originates from a self-selected subset of respondents who chose to provide free-text comments. This may introduce self-selection bias, potentially limiting the generalizability of the findings. Notably, in our study, those who used GenAI tools were more likely to have responded to the open comment section, and this likely influenced the valence of responses, potentially in a direction that was more positive toward AI. In addition, the web-based survey limited the response length and the opportunity for more nuanced insights. As noted, our thematic coding could be considered as resulting in high-level categorization of surface-level views rather than a deep interpretive analysis of participants’ underlying motivations or complex experiences. Finally, the survey provided a definition and examples of GenAI tools (eg, ChatGPT [OpenAI] and Gemini [Google AI]); however, respondents’ interpretations of the term may have varied based on their individual experiences and familiarity with AI technologies—a common challenge in survey research on emerging digital tools.

While participant validation was not feasible due to the anonymous survey design, we observed strong repetition of core ideas across responses, suggesting thematic saturation despite the brevity of comments. In addition, the qualitative themes identified here are broadly consistent with the quantitative findings from the same survey (reported in a separate manuscript currently under review) [18]. Although that manuscript is not yet published, this alignment provides indirect support for the trustworthiness of our interpretations.

We recommend ongoing qualitative research to explore GPs’ perspectives on GenAI. More robust, nationally representative surveys are needed to provide a clearer understanding of physicians’ experiences and concerns. To gain deeper insight into GPs’ use of GenAI tools, future research should incorporate semistructured interviews. This qualitative approach could help to uncover nuanced motivations, perceived benefits, and concerns that may not emerge from online survey data alone. In addition, integrating objective measures, such as time-motion studies or electronic health record analytics, could better assess GenAI’s practical impacts on workload and clinical decision-making. Such comprehensive studies would provide a richer understanding of GenAI adoption, informing strategies to optimize its integration into primary care practice. Beyond these recommendations, we strongly emphasize the importance of extensive survey research to explore patient perspectives on the benefits, risks, and opportunities associated with these tools, including how they perceive physicians who use these tools.

### Conclusions

This study offers timely and valuable insights into GPs’ perspectives on the role, impact, and limitations of GenAI in primary care. A dominant theme among GPs was their limited experience and training with generative and ambient AI, despite recognizing its potential benefits for clinical documentation. Beyond this, GPs expressed ambivalence and concern about the broader impact of GenAI in primary care. Many were skeptical of its value and implications for their profession, while others remained uncertain or cautiously optimistic about its potential to transform health care.

Now, 2 years after the widespread introduction of GenAI, the ongoing lack of understanding and training among GPs remains a critical concern, potentially hindering effective and responsible implementation. Addressing these gaps through better, targeted education and structured integration efforts will be essential to harnessing the full potential of GenAI tools in primary care. Future research, particularly more extensive qualitative studies, is needed to provide a deeper, more nuanced understanding of GPs’ evolving attitudes about, and experiences with, GenAI.

## Supplementary material

10.2196/74428 Multimedia Appendix 1 Survey.

10.2196/74428 Multimedia Appendix 2 Informed consent.

10.2196/74428 Multimedia Appendix 3 Detailed information about the variables and their categories.

10.2196/74428 Multimedia Appendix 4 Raw qualitative data.

10.2196/74428 Checklist 1 CHERRIES (Checklist for Reporting Results of Internet E-Surveys).

## Abbreviations

AI: artificial intelligence
CHERRIES: Checklist for Reporting Results of Internet E-Surveys
GDPR: General Data Protection Regulation
GenAI: generative artificial intelligence
GMC: General Medical Council
GP: general practitioner
LLM: large language model
NHS: National Health Service
TAM: technology acceptance model
