# Supplementary material for: Generative Artificial Intelligence in Primary Care: Qualitative Study of UK General Practitioners’ Views
Source: J Med Internet Res. 2025 Aug 6;27:e74428. doi: 10.2196/74428 (PMC12327960; doi:10.2196/74428)
Supplement: Multimedia Appendix 2 [file jmir-v27-e74428-s002.docx]

# Informed consent

# Generative Artificial Intelligence in Primary Care: A Mixed Methods Survey of the Views and Experiences of UK General Practitioners

**L1**

Researcher name: Dr Charlotte Blease
Contact: charlotteblease@gmail.com
Title of Research Project: Generative Artificial Intelligence in Primary Care

You are invited to participate in a research study conducted by the University of Basel into general practitioners’ opinions and experiences with ChatGPT and other generative AI tools. If you decide to take part in this study you will be asked to answer questions on your experience, and opinions about ChatGPT in primary care.

This study has received ethical approval from the University of Basel, Switzerland (approval ID: #030-24-1).. Taking part is entirely voluntary and before taking part you will be asked to complete a consent form. All of your answers will be completely anonymous. In the survey we will not ask for your name or any personal details about you such as contact details. The information you provide will be identified by a numerical code. The survey will be managed by www.doctors.net.uk This survey is funded by the University of Basel, Switzerland and the University of Uppsala, Sweden.

The study will begin with some questions asking for your awareness and use of different generative AI tools such as ChatGPT. This will be followed by questions asking for your opinions/experiences about how these tools might affect patient care, your practice, and your job. The survey will close with some brief questions about your current employment and demographic questions (your age, gender, year when you started practicing medicine). Most questions will ask you to answer by choosing from options, some questions will ask you to insert a number (e.g., year when you started practicing as a GP, number of hours worked per week). The survey also includes an open comment box question.

The questionnaire will take around 3 minutes to complete and all participants completing the survey in full will receive 1,000 eSR points

If at any time you change your mind and decide to abort the study you can do so. Both partial and completed responses will be saved; but partial responses will later be deleted for the purposes of data analysis.

If you decide to take part in the study, you will help improve understanding of the medical community's views about sharing online access to patients' health records. At the end of the study all of the completed surveys received will be used in the publication of a paper in a scientific journal. Details published will be anonymous

You can contact the Principal Investigator is Prof Jens Gaab at any time if you have any concerns or questions about the study Contact details are: if you change your mind and would like to withdraw you can also contact [www.doctors.net.uk](http://www.doctors.net.uk) directly: dpo@m3.eu.com If you wish to be kept informed about the results of this project you can contact Prof Jens Gaab.

Contact details will be provided again at the end of the questionnaire.

If you decide to take part in this study, we appreciate your time and thank you.

Consent: To take part in the survey, please confirm you agree with all the following statements.

- I confirm that I have read and understand the information supplied above for the study (1)
- I understand that my participation is voluntary and that I am free to withdraw from the study at any time without giving reason (2)
- I agree to the use of anonymised quotes in the reporting of the findings (3)
- I understand that all the study data and documentation whether hardcopies or electronic will be kept for up to 10 years and will be disposed of securely if it is confirmed that they are no longer required (4)
- I agree to take part in the above survey (5)
- No (6)
