# Supplementary material for: Generative Artificial Intelligence in Primary Care: Qualitative Study of UK General Practitioners’ Views
Source: J Med Internet Res. 2025 Aug 6;27:e74428. doi: 10.2196/74428 (PMC12327960; doi:10.2196/74428)
Supplement: Multimedia Appendix 3 [file jmir-v27-e74428-s003.docx]

**Appendix 3. Detailed Demographic and Practice Characteristics of UK General Practitioners by Free-Text Comment Submission in a 2025 National Survey on Generative AI**

| Variable | Category | Overall  n=1005 | Submitted comments  n=611 | | Did not submit comments  n=394 |
| --- | --- | --- | --- | --- | --- |
|  |  | n (%) | | n (%) | n (%) |
| Role | GP Partner / Principal | 439 (43.7) | | 246 (40.3) | 193 (49.0) |
|  | GP Registrar | 36 (3.6) | | 25 (4.1) | 11 (2.8) |
|  | Locum GP | 150 (14.9) | | 98 (16.0) | 52 (13.2) |
|  | Salaried GP | 380 (37.8) | | 242 (39.6) | 138 (35.0) |
| Gender | Female | 486 (48.4) | | 324 (53.0) | 162 (41.1) |
|  | Male | 506 (50.3) | | 278 (45.5) | 228 (57.9) |
|  | Prefer not to say | 13 (1.3) | | 9 (1.5) | 4 (1.0) |
| Age | > 46 | 546 (54.3) | | 334 (54.7) | 212 (53.8) |
|  | < 46 | 459 (45.7) | | 277 (45.3) | 182 (46.2) |
| Practice place | Large town/city (e.g. Nottingham, Cardiff) | 154 (15.3) | | 87 (14.2) | 67 (17.0) |
|  | Major conurbation (e.g. London, Glasgow) | 191 (19.0) | | 133 (21.8) | 58 (14.7) |
|  | Medium town/city (e.g. Worcester, Dundee) | 230 (22.9) | | 140 (22.9) | 90 (22.8) |
|  | Small town/city (e.g. Thetford, Omagh) | 313 (31.1) | | 187 (30.6) | 126 (32.0) |
|  | Village/hamlet | 108 (10.7) | | 56 (9.2) | 52 (13.2) |
|  | Other (please specify) | 9 (0.9) | | 8 (1.3) | 1 (0.3) |
| Practice Size | > 10,001 | 526 (52.3) | | 330 (54.0) | 196 (49.7) |
|  | <10,001 | 479 (47.7) | | 281 (46.0) | 198 (50.3) |
| Used GenAI to assist clinical practice | No | 756 (75.2) | | 438 (71.7) | 318 (80.7) |
|  | Yes | 249 (24.8) | | 173 (28.3) | 76 (19.3) |
| Practice could be affected by GenAI | Decrease my risk of having legal action taken against me | 91 (9.1) | | 51 (8.3) | 40 (10.2) |
|  | Don't know | 387 (38.5) | | 235 (38.5) | 152 (38.6) |
|  | Increase my risk of having legal action taken against me | 280 (27.9) | | 172 (28.2) | 108 (27.4) |
|  | Neither decrease nor increase my risk | 247 (24.6) | | 153 (25.0) | 94 (23.9) |
