# Supplementary material for: Generative Artificial Intelligence in Primary Care: Qualitative Study of UK General Practitioners’ Views
Source: J Med Internet Res. 2025 Aug 6;27:e74428. doi: 10.2196/74428 (PMC12327960; doi:10.2196/74428)
Supplement: Multimedia Appendix 4 [file jmir-v27-e74428-s004.docx]

**Appendix 4**. De-Identified Qualitative Comments from UK General Practitioners in an Online Survey on Generative AI in Clinical Practice

|  | I am scared of the whole concept and its implications to GP jobs in future |
| --- | --- |
|  | sceptical |
|  | some initial anxiety but I am sure will improve overall care |
|  | AI cannot replace the spidey sense of a GP to know that something is wrong. |
|  | Great for recording patient consultation notes |
|  | no experience of AI. May be more helpful for a junior doctor or nurse prescriber. |
|  | So useful for policies at the moment. And teaching. |
|  | I can't believe that it is taking so long to integrate AI into healthcare. It is very obvious that this is the way forward, and would save so much time and manpower. Our work is algorithm driven anyway. |
|  | So far I have only seen significant clinical events happening due to AI clinical diagnosis. |
|  | AI will be a new tool to aid diagnosis, help with treatment planning and reduce admin burden |
|  | No experience |
|  | I haven't used AI. |
|  | I don't really know much about it |
|  | helping with workload |
|  | It may be a useful assistant but cannot replace the genuine interest, empathy and intuition of an experienced clinician. |
|  | useful to speed up documentation |
|  | patients and gp practices will start to rely more on the AI instead of the doctors |
|  | It helped me with communication and good language with the patients as English is not my first language |
|  | Only started using recently but I think this will exponentially increase over the next few years |
|  | I think I need more experience in using it |
|  | Have not had any experiences with using AI at work |
|  | Helpful for creating personalized treatment plans e.g. exercise plans |
|  | I think it's important to not just rely on AI and to use experience as part of the consultation |
|  | I hope that I will see some agreed national guidance on the use of AI in clinical practice |
|  | Helps in blood tests that is normal -decreases work load -helps in documentation-on contrary no empathy no plan generation - |
|  | I have little experience but I feel if we have some training in how this would be helpful in healthcare then I may be more comfortable with it |
|  | dangerous and not helpful as triaging tool at the out of hours service |
|  | Ai will aid diagnosis accuracy |
|  | Major problem in Scotland to progression is outdated software not supported and cannot use modern Ai or apps with it |
|  | Patient safety and confidentiality is a massive concern if AI comes into the healthcare sector, particularly in General Practice |
|  | to be honest ai not necessary in our domain |
|  | Generative AI has been an good asset in my work |
|  | Quick and creative solutions, helping to refine ideas |
|  | nothing for now |
|  | help me to solve some problems |
|  | I see that ai not important in our work |
|  | I will try it soon |
|  | AI help me so much |
|  | could be the end of medicine as we currently know it |
|  | Not enough experience of this area to comment |
|  | might lead to improvements |
|  | as a scribe, it works well but I wouldn't use it for diagnosis |
|  | AI tools need to be tested, validated and funded if expanded use required |
|  | I think it can be helpful but definitely needs checking by a clinician and there may be unintended consequences |
|  | i am keen to try as my colleagues have reported success |
|  | early days yet |
|  | AI has to be very carefully implemented |
|  | Need more knowledge |
|  | I am not convinced AI will help |
|  | I have not used it yet, it worries me about being able to spot it use, in terms of medical students/GP trainees for academic work |
|  | not much expereince in this yet |
|  | Interested to potentially use it in the future. |
|  | I have concerns about data security with AI use |
|  | It is affects role as GP trainer especially trainees eportfolio |
|  | I feel AI will not read patients and miss the social cues and hidden agendas px have. Px will say what they think they should rather than a doctor responding to non verbal cues |
|  | No experience in this at all but aware of it |
|  | aI IS APPOWERFUL TOOL IF ITS HARNESSED WEL. IT IS DIFFICULT TO MOVE AWAY FROM THIS EMERGING TECNOLOGY BUT NEEDS PROPOER TRAINING IN ITS USE FOR UNDERGRADUATES |
|  | Generally a lot of obstacles seems to be put up by my practice to using generative AI |
|  | our practice will certainly change. accurate AI diagnostics ought to massively decrease the attendance of the "worried well" as long as it doesnt function like 111 which massivly increases the attednace of the worried well! |
|  | I am sceptical of any potential benefit in current NHS environment and believe would lead to increased inequalities in care and worse patient outcomes overall. |
|  | Helpful tool but doesn’t replace our role as GPs |
|  | I feel they are useful and do not diminish the role of the physician which others unfortunately feel |
|  | Will help a lot in administrative tasks |
|  | Am happy to try and plan to use AI more. |
|  | if AI makes a mistake I imagine the clinician will be responsible |
|  | not yet but no doubt on its way |
|  | No thoughts |
|  | Concerns |
|  | I do wonder if we as a society are mature enough to understand what this all means |
|  | I don’t know much about it |
|  | Only had presentation from Heidi. Not interested , document too much safety netting/safe medicolegal notes not useful to read back by another healthcare proffessional. I write a shorter , clearer note , and am processing/deciding information as I write them not as I speak in consultation. Referral letters would likely be rejected from sending AI summary |
|  | It is useful and should be encouraged |
|  | will increase GP workload |
|  | I don't use it, AI cannot replace experience and context of care |
|  | Have used to write reports and documents. Could help with generating specific advice and care plans |
|  | I find that using Heidi intermittently will significantly reduce my documentation time. You do need to read the transcription prior to use in the notes and you do need to input your observation findings to allow for coding but otherwise i find it very useful especially with more complex histories or mental health patients who may take longer consulting times. It also means that i am better able to maintain eye contact with my patients rather than turning away to document which i think improves the patient relationship. |
|  | Have used AI dictation software and found it very helpful |
|  | We are currently prohibited in having third party software |
|  | Very worried about the clinical safety. Also concerned that it will over diagnose (like ‘dr google’!) |
|  | I hope that it will reduce the burden of admin to allow more time dealing with patients |
|  | i haven't used generative AI yet, still studying it |
|  | Currently don't it or have access so feel would need training and more experience in use before giving an opinion of it. |
|  | Have no experience of AI but will look into it |
|  | To explain AI results |
|  | Many thanks, very interesting |
|  | Very unsure. Errors are easy and may be missed |
|  | Useful to explain concepts |
|  | Has been helpful to some extent |
|  | only used briefly, Heidi for note taking, |
|  | GPS have no training in AI. I would be happy to get involved but have no experience |
|  | I don't think it will be helpful for every aspect of gp |
|  | Cautiously positive about the potential for AI but too early to say and also concerned about its impact on job security and the role for some specialities |
|  | concern re. unsteady future for GPs?? |
|  | More training requires |
|  | I need to understand more |
|  | I dont know much about it |
|  | I have minimal experience of this |
|  | I believe AI is a Pandora’s box that has already been opened. We lack the safeguards or infrastructure to control its possible (and potentially catastrophic) effects. AI cannot replace a doctor, on many occasions (daily) I have picked up on important subtle cues from a patient (posture, pause in snswering, stutter, miss placed word, subtle sigh, change in manner) that entirely changed the scenario. No checklist, pathway, algorithm or AI could do this. |
|  | AI will evolve and does have a medical role |
|  | I don't have enough experience really. I'd like to use AI for note taking during consultations but difficult to implement in terms of privacy, confidentiality, security. |
|  | not used but curious |
|  | I am rather suspicious of the use of AI in genearl practice. Maybe more suitable to other hospital specialties? |
|  | Particularly helpful in designing teaching presentations for trainees |
|  | My experience has been in filing results and registering patients, it is good but we are still trialling at the two practice I work (one has been using it for longer and seems to work very well) |
|  | AI will help but no replace |
|  | I think AI can be useful in some circumstances. Like in informing patients of normal results, contacting then for routine follow up etc. And for giving information for coughs and colds. I would not want to rely on AI for other diagnosis use |
|  | No experience of this |
|  | Interested in the potential use of AI to document consultations and treatment plans. |
|  | I am not sure it will ever replace “the human touch” |
|  | total inexperience with it and not well informed on it |
|  | Will still need personal medical overview |
|  | AI is only as good as the data input. I personally use AI systems elsewhere to generate initial letters or descriptions. However sentiments expressed and empathy are often inappropriate and excessive and need a human review. WRT AI generated letters we already have hospital generated letters which are frankly 4 sides of A4 crap detailing nothing we want to know where we struggle to find the salient points ie diagnosis, hospital treatment and follow up plan. |
|  | I suspect a patient might present with a printout of a management plan suggested by AI which may or may not be appropriate |
|  | AI |
|  | I expect increased usage of AI tools will increase requests for testing due to defensive practice from AI and the doctors using them. |
|  | Might be helpful to make things more efficient |
|  | not really interetsed |
|  | the great unknown for me |
|  | I don't have personal experience of this, so I'm not clear on the advantages and disadvantages. |
|  | no experience yet |
|  | May be used for filling in appraisals |
|  | I have never used it |
|  | Have used in research with mixed outcomes |
|  | I don't know enough about it to be able to comment |
|  | I am fearful but think it could be useful |
|  | Using Heidi as an ambient ai scribe has made a massive improvement to my working efficiency and quality of notes |
|  | Using this for note taking means that I can look at the patient more, it means I have more time with them as I do not have to account for documenting |
|  | I don't think this would be of use |
|  | I think could be incorporated |
|  | Patients need to see and hear a highly trained professional not a computer. |
|  | I need more guidance on suitability for general practice in my location |
|  | Helpful with explanations and understanding things too |
|  | Not yet used but I think it could be a massive help |
|  | Its not being used in my GP practice at present but I think its only a matter of time |
|  | AI is important if used correctly |
|  | Uncertainty as general practice is about superb communication and risk sharing |
|  | not keen |
|  | None identified |
|  | I think it greatly improves documentation but we were banned from using it due to GDPR concerns |
|  | Need much more data about its use and statistics going forward to decide on the good and bad points. |
|  | I have limited experience in this area. It has a place in some circumstances, however my concern is that it will be used to try and replace clinical staff, rather than support them, as with so many initiatives in the past. The result is then less clinical staff forced to do more and technology often remains inconsistent and unhelpful. |
|  | Cautiously interested |
|  | no idea on the subject |
|  | i have colleagues who use dictation technology using AI which i would like to explore |
|  | It’s good for patient communication |
|  | A recent study showed that chat GPT was more empathetic than doctors in dealing with patients. Also, chat GPT managed to pass AKT exam for GPs. So I certainly think that 111 triage and other roles like that, which are algorithm-driven, will be done safely by AI without risk of stress or burnout. But it will take time to evolve. I am all up for technology. |
|  | I worry that management will be seduced by the idea of a technological fix to workload and waiting lists and roll out an expensive solution which hasn’t been tested and doesn’t actually solve problems or address real world problems |
|  | We just trialed Heidi to help with notes, I don't use it much as not very helpful. I do not have an employer, I am a GP partner. |
|  | Have not used it. |
|  | I think these technologies are in the early stages of development. The next few years will see more powerful tools |
|  | dont like AI |
|  | think it can be a super helpful tool if used in the right way and has huge potential to increase efficeceny in GP |
|  | about to use for summarisation of consultations which I hope will be helpful |
|  | i have not seen any AI tool yet in my patch |
|  | I am aware my practice is currently looking into AI documentation software. |
|  | very useful for meetings and writing up minutes |
|  | I have not experienced it |
|  | Not sure |
|  | It will dehumanise medicine |
|  | Very new concept |
|  | app designed by collleague is excellent and extremely useful |
|  | GAI not used in clinical practice |
|  | I don't want to use AI |
|  | No experience of generative AI |
|  | Colleagues find it useful but I have not tried it yet |
|  | No experience yet |
|  | nil to add as not yet used |
|  | Find it good for note taking and I guess about 90% accurate |
|  | completely changed my enjoyment of job - means I can focus on pt rather than busily typing away |
|  | This is here to stay and if anything, have increased presence in our working life. So we as clinicians need to embrace it! |
|  | A lot of negative misconceptions about AI in medical practice |
|  | AI works with algorithms. It is useful but cannot replace human judgement and emotional connection. |
|  | good for admin related not clinical |
|  | looking at them , like Heidi |
|  | No experience of AI |
|  | More info about what's available to use |
|  | It could improve the care from substandard clinicians. |
|  | Think there are lots of opportunities. Hope it’s introduced in the right way |
|  | Too many billionaires controlling our work as it is. Stuff AI doing turning us into robots. |
|  | Dermatologist locally uses it |
|  | we dont use AI |
|  | No experience |
|  | cautious approach needed |
|  | good for non clinical stuff, summarizing long documents ie contract stuff, writing reports for staff and other non clinical admin |
|  | Not sure our patients will like this. |
|  | I've had little exposure so far but I don't think it can replace the core of my work and doubt it will add significantly to my own efficiency |
|  | I have not used AI as yet |
|  | Really useful for documentation of long consults |
|  | no experience of generative AI |
|  | No additional info |
|  | Likely to put more pressure on gp |
|  | I have no experience of using generative AI as a doctor but would be concerned about is use in diagnostics as a patient in the future |
|  | not had any experience with AI tools |
|  | I have not had any training in this so unaware what it can offer |
|  | Not used yet |
|  | will get a lot of worried well and over investigation |
|  | I would be very surprised if AI does not completely take over the role of GP’s over the next 15 years. |
|  | Good for notes but otherwise too much unfiltered date. Not holistic |
|  | I have not used AI in my clinical work yet. |
|  | dangerous in it's current form |
|  | still relatively early days in my usage. think may get more benefit from transcription in notes for use in GP. |
|  | Ethical considerations have to made in consideration for the usage of ai tools in general practice |
|  | No personal experience but creates problems with clinical risk as there will be inevitable errors |
|  | If it works quickly in Microsoft might help |
|  | know very little about it as yet |
|  | nil else |
|  | I do not have direct experience of AI in my work, but am aware of this being a thing in the near future. |
|  | I feel the personal aspect from patient care is removed and have concerns about vulnerable and elderly patients who may struggle to access/benefit healthcare due the AI |
|  | good for documentation wouldn't use it for clinical reasoning |
|  | No experience |
|  | Early days still in GP. Very useful in teaching |
|  | I have a colleague who uses generative AI to write letters. This is my only experience of generative AI being used or spoken about at all in healthcare. |
|  | Have no experience in using them |
|  | Low level AI can be useful. Not all patients will benefit or be able to access this. |
|  | No experience |
|  | No experience |
|  | transcription software integrated within clinical systems (eg EMIS) would really save time and improve patient contact time. AI could be used to really improve the efficiency of primary care. It can also improve safety but suggesting possible differential diagnoses. |
|  | Limited at present |
|  | To be handled with care- typing autocorrect systems for example often change documents inappropriately ,meaning you have to read carefully before saving. |
|  | Concerns re errors |
|  | Will be the future but not well incorporated into software such as system one yet |
|  | Heidi occ used for long referral letters |
|  | Recent articles on how AI changes actual information given continues to be a concern |
|  | Like All things new there is uncertainty but with time we can adapt it to benefit |
|  | So have have mainly used AI for general communication with allied Healthcare professionals / consultants. |
|  | In surgery will start using it |
|  | Usually I am using in iPhone,but would be using in the Surgey computer |
|  | AI is great to find answers and literature for very specifc questions |
|  | ai seen as the new dr |
|  | It is quite pragmatic, with no empathy. |
|  | I am hopeful that it will aid a great deal with documentation/administrative work |
|  | I'm sceptical of its use and feel its likely to significantly reduce the demand for GPs |
|  | Keen to use for documentation. |
|  | It is a good tool for drafting letters, summarising things, communicating with others. But it has got its limitations. I’m not sure how good it will be in diagnostic accuracy and I doubt it will be any good in showing empathy towards Patient and stuff like that. |
|  | Significant concerns regarding privacy and data protection |
|  | will it be a tick box activity |
|  | I have no experience in this area thus far |
|  | Increase queries by young generation |
|  | No experience of it as not true Heidi |
|  | no experience as yet |
|  | No insight into it at all |
|  | Extremely helpful in the main. Can use to get answers to questions that i might of needed to phone a hospital colleague about in the past |
|  | Anxious about its use. Would need a lot of training and to understand the data risks associated with it. |
|  | i don't have anything specific to add. I don't have much experience of using generative AI in my work. I can see that there could be potential benefits, but there are also downsides and i think the value (or otherwise) of generative AI depends on how it is utilised. |
|  | I know very little sorry |
|  | Patients will navigate AI systems to get the end result that they want |
|  | We also have AI assisted triage and didn’t find it useful |
|  | limited experience |
|  | Think Healthcare roles will adapt as used more |
|  | I would be interested in generative AI if it reduces my workload. |
|  | I use co-pilot during consultations to get rapid access to information that I know exists but cannot recall |
|  | No experience to date but very excited by this development |
|  | AI has huge potential but also huge risk |
|  | Clinician using AI is the future. |
|  | very limited experience |
|  | found it helpful to politely correspond wiht secondary cre when I am not as good a written communication and can come off as passive agressive, CHATGP prevented this. |
|  | AI is open to abuse |
|  | Still concerns over third parties having access to patient data. |
|  | No experience |
|  | Generally good to use AI to triage patients but still needs an overall review of cases as some urgent cases are triaged as routine and vice versa. Overall, it has helped. |
|  | I have no experience yet, I can see benefits in provision of information but I think the gains from interpersonal interaction are being underestimated |
|  | Not yet started using but have concerns about building reliance until effectiveness and accuracy really known |
|  | I don’t know enough about this |
|  | It’s less about the tools more how they are regulated, governed, resourced and implemented. Like any digital innovation - it has the potential to both improve and constrain work. |
|  | Still a work in progress |
|  | we make judgements esp our ability to deviate from guidelines/protocols etc is what makes us so cost effective |
|  | No exposure in my work |
|  | Can make mistakes |
|  | Potentially useful but may lead patients not to seek GP advice ..could be dangerous |
|  | not yet exeperienced |
|  | Never used iAI in any arena |
|  | Due to start using thus unable to give any opinion as yet |
|  | I think cpuld help with non clinical elements |
|  | Still early days and can't be relied upon yet |
|  | not accurate enough to really help now = too many errors and needs checking |
|  | I don’t feel comfortable working with AI. |
|  | Lots of work still to do in this area |
|  | It will be transformational for healthcare |
|  | Have found useful for complaint responses, summarising etc |
|  | Theory sounds good. Dangerous in reality |
|  | I haven't had the opportunity to use this yet |
|  | I haven’t used it but colleagues have used for letters and found it helpful |
|  | I have not used for direct patient care yet as I do not yet trust the accuracy of this |
|  | I don’t have experience using AI at work but it will surely be a helpful tool to help healthcare professionals. I am thinking as a GP, AI could help me with admin work so I have more time to focus on clinical work. |
|  | This is new ground for me. I have not tried it and am not rushing to do so |
|  | No experience of AI |
|  | NHS NEEDS TO LINK ALL IT'S ELECTRONIC RECORDS |
|  | Helps with language for writing reports |
|  | I use it for policy drafting and writing |
|  | Felt it was helpful when looking for some specific information but of course check others nhs guideline |
|  | All very unknown to ke |
|  | No experience |
|  | I don’t feel i have adequate information or knowledge about practical applications ocAai |
|  | I have had little use of AI but I see it will be an area I need to learn more about |
|  | I have not had much experience however I do expect to have more experience in the future |
|  | I don't have personal experience but very interested in this area as I feel it will expand rapidly |
|  | I am concerned that it will become increasingly self referencial |
|  | Highly recommend ANIMA as medical AI |
|  | Useful tools, but tools only |
|  | unsure if will help |
|  | might decrease workload but also decrease the job opporunities |
|  | No experience of generative AI professionally or otherwise |
|  | I have never used AI but have concerns about its use |
|  | I have never used it and am not aware of his it could be used safely |
|  | Patient expectations are already high but will be raised exponentially with AI |
|  | More reliable AI tools for data protection |
|  | I will need more training |
|  | It is an evolving field, which I think will only get better. |
|  | Uncertainty |
|  | Not much empathy in responses, sounds fake |
|  | allows me to focus on patients rather than my note keeping |
|  | AI is not specific enough to understand nuance of practice and does not have that gut feeling or sixth sense that comes from experience |
|  | Patient demand will increase. |
|  | I don’t know what to think about it, our job is nuanced |
|  | Have little experience with AI |
|  | I think we are being left behind in general practice when it comes to using AI. |
|  | Options did not include c the signs , patches , AccuRx |
|  | not convinced about it's use for clinical practice- but am sure it is the way things are heading. did find it useful when taught how to use it to plan a tutorial for trainees |
|  | none so far, but we will hopefully be using AI thi coming year in an offical capacity with central funding |
|  | concerned about job security |
|  | Apprehensive but acknowledge the need for it |
|  | Not enough knowledge about it |
|  | AI does scare me going forward |
|  | I have concerns about who will regulate the provision of AI tools, and what impact their use will have on liability for medical errors |
|  | Not used it |
|  | Doctors need more training in using AI |
|  | It’s something I’ve not used yet! |
|  | AI will be a useful tool in future medical practice but may not replace the F2F interaction between the clinician and the patient. the |
|  | if AI comes and takes over the diagnostic decision making from GPs, then we all shall loose our jobs. |
|  | Very useful and almost accurate |
|  | we experimented with a system called ANIMA and had to abandon it |
|  | Only use is self taught so far but I suspect there will be an explosion in next couple of years. |
|  | I m concerned about confidentiality and accuracy of information. |
|  | i have no experience of AI in my work |
|  | not used before |
|  | Don’t know enough about the tools available of how patients are using them. Aware of colleagues generating notes or letters with assistance of them. Aware of practice manager running letters or responses to complaints to help draft letters. |
|  | Trialling Heidi health transcription tool too |
|  | I dread to think of human doctors being replaced by AI |
|  | the ability of generative AI to " hallucinate " is alarming |
|  | Dangerous |
|  | It's not good enough yet to use for diagnosis, but very helpful for rewording things. Therefore not reducing time spent yet. |
|  | Very suspicious of AI, it depends totally on quality of information inputted. Who is medicolegally responsible |
|  | Patients already use it and sometimes it can be useful but depends what and how the question is asked |
|  | No experience of using so far |
|  | we use anima AI program to assist with triage- but i think still needs clinical oversight |
|  | I have found it very helpful and am excited to use AI more in my work in the future |
|  | I hope I get to retirement before all this nonsense takes over |
|  | I feel very sceptical about the benefits of AI as a replacement for real human interaction when this matters. We are creatures, not robots. |
|  | Have heard of Heidi for documentation but have no more knowledge of it |
|  | Does allow more concentration on the person in front of you rather than documenting as you go |
|  | none |
|  | AI is never a panacea - may be a useful tool if programmed properly and thoroughly - no GIGO please (GIGO = Garbage In, Garbage Out) |
|  | i think AI benefits are overstated |
|  | Not great for human contact |
|  | unsure of it role yet |
|  | I have no experience with AI |
|  | I use AI in my non-clinical work; but do not feel it is safe or ready to be used in clinical settings safely. |
|  | Not used much |
|  | 'Dr Google' already undermines much of the info or advice we might share with our patients. Don't want to become a prescription vending machine. |
|  | I have no experience of AI |
|  | Early days for AI in medicine. Interesting times ahead. |
|  | The only experience I have is in the auto filing of pathology results so far |
|  | Used by one of our partners for documentation |
|  | I have had little experience of AI in my work,and have reservations about its use in a clinical setting |
|  | Have heard but not used patches ai and others using chat gpt |
|  | not tried it but have had reasonable things |
|  | not experience AI at work |
|  | I find it helpful |
|  | Very important that understand what generative ai can and can’t do- proper training in use is critical |
|  | I am still trying to understand it |
|  | Seems like a really positive way forward. Need to be sure about ethics re info sharing |
|  | I see use amongst my colleagues at other surgeries mainly in dealing with complaints. |
|  | Very limited experience |
|  | i have very little experience of using AI |
|  | one issue is setting time aside for training in this |
|  | Concerns re AI induced errors eg Apple News false information. AI hallucination needs addresses |
|  | Generally speaking it has not saved time because of post 'editing' of generated text |
|  | Would like to be more involved. |
|  | No experience |
|  | useful and saved time |
|  | Unsure |
|  | No experience |
|  | Not used AI yet. |
|  | Patient satisfaction may decrease |
|  | GP is not a role that should be undervalued. |
|  | Identification of what can be introduced while also maintaining patient safety |
|  | The biggest issue is voice recognition software accurate enough to follow a patient consultation |
|  | no experience |
|  | In a healthcare system where there is never going to be a) the money and b)the personal investment in changing the ways we work as humans and making the system and environment a workable place for humans, AI tech is going to be necessary to reach more people and try to offer a half decent healthcare. But, my experience of AI being used in the commercial world eg banking etc is that yes it makes things more efficient, I can do things at 3am in the morning, but it's definitely more impersonal and some people will like it and some people (including those who are tech savvy) will hate it. The older people on the whole will not see it as an improvement and will feel uncared for. |
|  | I dont intend to use |
|  | The effect of AI depends on how it is implemented and the rules surrounding its use. There are many risks and many potential benefits and good regulation will be key. |
|  | AI is useful and must have |
|  | I think AI is the way forward |
|  | Not much experience...but it takes away gut feeling |
|  | Will aid diagnosis and results, coding. May provide barriers between Dr and patient |
|  | have not been told enough as yet |
|  | There’s no stopping it now |
|  | i welcome use of AI in diagnostic testing and diagnosis but have had no info or training |
|  | I have seen a consultation documentation AI app - found it helpful but still in learning mode for it |
|  | Not used it in work have used outside work |
|  | I really don't know much about it & have never used it |
|  | I am pro AI but don’t have enough time to develop it |
|  | it helps to edit anonymised reports |
|  | It needs exploration |
|  | limited experience, I just don't think that patients will usr it appropriately |
|  | currently limited use and need further training |
|  | I had an extremely positive experience with AI with my referral and less time spent by me so had extremely helped me , I wish it could be intergrated into the cliincal systems asap |
|  | Potentially going to change the face of GP |
|  | Use of AI will help in admin and documentation which is major chunk of general practice |
|  | I really don’t have any experience |
|  | I think it will be used to reduce workforce quality. |
|  | Good for doing letters and complaint responses. Not used in consultation yet |
|  | AI is useful providing that there are set parameters. Patients will not necessarily use the right terms, and AI will not necessarily understand patient phrases or pick up on the degree of a symptoms. Therefore any AI differential diagnoses need to be taken speculatively. |
|  | Needs more information on how AI can help. |
|  | I don't have any experience with generative AI and would be hesitant to use it without a significant amount of training tailored to a GP setting. |
|  | Needs more exploration into it together with appropriate training |
|  | No experience so far. |
|  | AI is a tool to help to become more efficient but if cannot and should not replace the doctor |
|  | I look forward to seeing the benefits of AI for improving patient care |
|  | Should be provided to all doctors as a free service. |
|  | would like training |
|  | The AI tools show promise but not quite there yet. |
|  | useful sometimes for admin |
|  | To generate interview questions |
|  | Nervous of it |
|  | Isn’t it the inevitable progress? |
|  | No personal experience. |
|  | not sure |
|  | I mainly use it to rephrase letters and messages. |
|  | Note summarising is a game changer in terms of admin and clearly more could be done by AI if systems were more integrated |
|  | Quick to present possible relevant information |
|  | be careful of AI |
|  | Not used or considered |
|  | It would be good to see specific examples of how AI can be used to assist clinicians. |
|  | Interested in AI use of note taking, letter generation and searching for information on diseases |
|  | no experience |
|  | Useful at point of NHS entry to standardise 111, OOH , GP and A/e |
|  | Very early to say. Lots of unknowns |
|  | Concerns re gdpr and accuracy have made us stop using heidi |
|  | I don’t have much experience with generative AI, I would like to learn more. |
|  | Complete I know to me really but it’s very topical |
|  | not keen |
|  | Heidi is fabulous. It saves me an hour a day but also a lot of brain ache trying to remember which finger or how many days a problem started. Importantly in the consultation I can focus more on what the patient is saying rather than focus on trying to remember everything which I was struggling to do. |
|  | no experience in the work setting, some in my private life particularly translation |
|  | Really no idea |
|  | still need to be convinced at any use other than machine diagnosis (eg radiology analysis) |
|  | Useful for non clinical tasks thus far |
|  | No experience in my work. Experience outside of work with inaccuracies of data from AI |
|  | No experience of generative AI |
|  | I don’t know enough about how this will be implemented or how quickly |
|  | Wary about the models these AI are trained on so my preference is for its use in dictation and composition of notes and letters. Pitfalls where salient info may not be captured as I’ve heard happening in a practice recently, waiting to see if there will be any detriment in that |
|  | Increased use of AI in appraisals seen |
|  | we use very little due to GDPR concerns, regulatory concerns, and do not use in direct patient care currently as a result of safety and appropriate use concerns, however would like to as aware increases efficiency of documenting for many drs. |
|  | No experience |
|  | It is very good at writing policies required for CQC. It is also not bad a first draft of complaint responses, to give a basic framework. |
|  | Have tried to use Heidi but ICB banned its use |
|  | Positive |
|  | I am going to try AI Heidi soon |
|  | Heidi AI good for transcribing consultations |
|  | Doctors with AI will replace doctors without AI |
|  | we will still need the human touch. AI can't do everything. |
|  | I have noted some colleagues have used it to reply to complaints, although it is presumptive and have not been able to confirm it. |
|  | technology is best used to enhance not replace the expertise and experience of an expert |
|  | No experience |
|  | Good tool but will decrease the need for f2f GP |
|  | Sceptical of many uses but sometimes it will be great |
|  | Nil experience |
|  | Useful for general information but less so for personalised use |
|  | some AI to help reduce paperwork/admin wqould be helpful |
|  | I don’t have experience |
|  | Its a force for good |
|  | unknown |
|  | AI cannot take away the personal touch, rapport and empathy |
|  | Not used but can see some potential uses |
|  | not used AI but would like to be introduced to it |
|  | I don’t know much about AI In healthcare at all. I think it could have benefits but also drawbacks and should complement medical services rather than replace them |
|  | Looking at getting programme to help us with results. Watch this space! |
|  | AI will just confuse thwe issues and will harm the elderly |
|  | can be a dangerous tool for the public |
|  | Limited experience as yet |
|  | Really limited exposure to AI in the workplace |
|  | nil as I have had no experience |
|  | I find it more helpful for getting through administrative tasks rather than clinical |
|  | Nothing to report. |
|  | total minefield and I have no knowledge in this area |
|  | i think these tools are great for comms - responding to complaints for example but nothing replaces clinical acumen from years of training and experience |
|  | It's very early to judge how effective and useful thay can be |
|  | If we use AI too much it will replace us. AI can not have "gut feelings" which are actually pretty important in clinical work (in my opinion) and removes any element of compassion/empathy. No patient really wants to be seen by a robot. Would you? |
|  | Needs to be done in a safe and measured way - uncertain how it will help with issues surrounding history taking |
|  | II haven’t used it yet |
|  | It is also useful to create policies and SOP |
|  | Know others that use for picies complaints etc can see this is ide |
|  | Data input must be of extremely high quality in order for generative AI to be meaningful |
|  | In early stages of its development and application in healthcare. |
|  | only used it for notekeeping but i think increases my ability to give the patient my concentration rather than notekeeping |
|  | I would like more training as I think it could be useful |
|  | I use ChatGPT frequently and it has enormous potential and is very useful but it needs sense checking and it could cause complacency in busy overstretched healthcare professionals. When you start using it it appears to have a solution to many problems and it also converses with you in a way that feels authentically human. These are impressive qualities but it should only be seen and used to augment rather than replace effort. |
|  | Not sure how helpful it will be for primary care, except in the case of helping in quick documentation. |
|  | Limited experience but privacy will be major concern |
|  | Will negatively affect patients |
|  | no idea |
|  | I still remember I robot - there are times in my profession I used my "GUT" and it has been pretty good to me so far. People do not fit into boxes so I think AI will work on probabilities, In I robot the robot chose to save will smith over the child due to probability that is not human and that is my fear of AI - sure useful in some circumstances but others not so much. |
|  | I haven't had the chance to use it yet |
|  | possible use for clinical notes taking |
|  | I am anxious that it be good enough to perform safely prior to implementation. Some patients will never embrace this. |
|  | I have yet to use but I'm not interested. Clinical judgement, human empathy and the insight and subjectiveness that has shaped the medical profession over the last 100 years cannot be replicated by AI, not now, not ever. |
|  | Still not confident it is safe and reliable enough for history taking/diagnosis/management but useful for admin/management eg policy drafting |
|  | generally helpful |
|  | There is no role of AI in my role. We need real, not artificial, intelligence to look after patients. |
|  | Apps like Heidi are being talked about locally for consultation |
|  | I have no experience in this area at all |
|  | very helpful for speeding up documentation |
|  | more training needed |
|  | Would be helpful for patients to use before contacting or seeking medical help |
|  | not used but its coming... |
|  | Great if used as an adjunct, can be very helpful, but also dangerous if not used appropriately. |
|  | I have heard of other Doctors using AI to generate letters for patients, but have not used it myself because I am worried about it taking away from my skills as a Doctor. |
|  | No experience if AI |
|  | Dermatology referral process using AI locally |
|  | No experience |
|  | Not used AI |
|  | have found it helpful to re-phrase content sent to patients to make it easier to understand or remove jargon |
|  | Not used AI so fat |
|  | it would be helpful when documentation. compiling response to complaint letters etc helping with website issues |
|  | I don't know enough about AI as don't use it much |
|  | I think it should not be used in practice. It will take away the human touch and clinical acumen. It is dangerous. |
|  | No experience of using generative AI. |
|  | If found beneficial there would need to be funding for AI and also any changes in medical defense fees. |
|  | it is a subject I am interested in learning more about as I have no experience in this |
|  | We use AI as part of anima triage system |
|  | Worry about indemnity if using |
|  | no experiences yet, but surely will come soon. |
|  | GP's are a dying breed. We had an informal discussion over our last Christmas meal if we need to now become IT professionals or even a bricklayer. |
|  | Good to define which areas it could be used for |
|  | No experience of AI, and I have not considered it's impact previously |
|  | Some doctors are using AI note scribing tool |
|  | It needs to be delivered with inclusive training so less IT confident colleagues are not left behind. It also needs to be carefully worked through to ensure that patients retain ownership of their own data unless they give specific consent to sharing specified items with a clear understanding of purpose. |
|  | we are considering the use of scribing which i think will help efficiency and accuracy of recording in notes |
|  | limited experience but I have concerns about the technology at present |
|  | I've found it useful for keeping up to date with latest guidelines and improving knowledge, to summarise conditions and management |
|  | If we do not engage with this we will be doing our patients and the health service a dis-service |
|  | Need far more training on AI use in healthcare setting than currently available. |
|  | Concern over accuracy |
|  | No experience |
|  | None, don’t know enough to comment |
|  | early days but i think will be useful |
|  | I've not yet considered using AI in its present format, but I remain open minded. I don't expect things to improve much though; our present problems are just too entrenched. Addressing demand, unrealistic expectations and personal responsibility are going to offer more hope, than gimmicky AI 'solutions'. |
|  | Very interested in exploring the use of AI in the role of a busy GP |
|  | Heidi Health has been incredible for generating excellent documentation for consultations that improve communication and reduce my workload as a GP. Very accurate with medical jargon used |
|  | Primary care does not need millions of pounds spent on AI but needs the current IT infrastructure to work efficiently and smoothly with seamless connection/working between systems within primary care and those belonging to other organisations. |
|  | As more people seek generative AI information so will the need to clarify what the information mean |
|  | I worry about how it would work feasibly without causing errors |
|  | I do not have any experience of AI in my practice but I am interested. |
|  | I was involved in the beginnings of an ai service as a tester and it failed miserably because patients aren't textbooks and it couldn't cope with the greyness. |
|  | Look forward to implement once approved |
|  | uncertain about its impact |
|  | will like to use AI |
|  | It has the potential to aid in management of patients but there is a long way to go for its diagnostic accuracy |
|  | no experience |
|  | Need more training to fully understand it |
|  | I can perhaps envisage using generative AI to improve efficiency of dictating or creating correspondence patients regarding certain generic topics e.g. ‘please dictate a letter to a patient explaining the link between their high cholesterol and cardiovascular risk and the treatment options’ or ‘ please dictate a letter to a patient explaining that their x ray demonstrates mild osteoarthritis of the knee with advice on management options’. However I am currently sceptical about the benefits of using AI during a consultation. |
|  | Still not widely used and unsure about it all |
|  | No experience but can imagine it could have a useful role as long as not used to substitute real doctors |
|  | It’s not ready yet but it’s coming. Manager uses it to respond to general complaints. |
|  | Saves time transcribing consultation and generating referral letters. Not perfect but helps |
|  | I have limited experience so far but see it as a positive if the whole team understand its role and how to use it. |
|  | I am concerned that generative AI can be skewed by inaccurate data, and with reduction of fact checking online, this may reduce the accuracy of AI. |
|  | Until licensed as medical device will be medico-legally challenging to implement use. |
|  | Need more proactive support and resource to take on such tools to help support general Practice utilise such tools - potentially transformative potential in day to day management and case finding. |
|  | Interested to see how it would be introduced |
|  | Heidi |
|  | I have not had personal experience but I do worry about the future role of AI |
|  | No experiences |
|  | it is an interesting area of development to watch. We need more understanding of how to use clinically and non-clinically. |
|  | if there are enough safety measure, AI would be much valuable |
|  | Not used it and frightened of it being implemented |
|  | no experience |
|  | Not had any known experiences with AI |
|  | I do not have any practical experience to add relating to generative AI |
|  | I have tried AI scribes but find that they don't save time. |
|  | Devalues Gp role |
|  | I'm unsure of how it will be of benefit |
|  | I don't believe AI will ever replace clinical acumen or the simple "gut instinct" of a trained GP. Nor will it be able to give the rapport/empathy that so many of our patients need and find therapeutic in itself. Hopefully in the future it will be useful as an aid to diagnosis but I think the legal ramifications of relying on it will need to be robust before it gets widely adopted. |
|  | its a quicker form of information when addressing a specific question |
|  | if used properly can be a force for good |
|  | i have not used this |
|  | no experience yet |
|  | We need more understanding of the concept. We should know where to draw a line. |
|  | Now comes up at the top of a Google search, so appears without actually seeking an AI answer. This makes it easier to access, while also diverting attention from website based results |
|  | I see these as a tool to help efficiency not quality of care |
|  | Could be developed further |
|  | Not used so await further guidelines |
|  | Somewhat apprehensive of AI |
|  | My work has discussed HEIDI (notes dictation AI) and is awaiting info from the ICB as to whether it is deemed sensible to trial |
|  | This will help in certain limit but as a GP this will create more work |
|  | useful to create teaching material |
|  | Love to see AI deal with some of my patients long winded consults about vague symptoms they have had for 20 years |
|  | I have not intentionally used it much at all - only the AI summaries when using Bing search sometimes provide helpful information, but at the moment I would not trust it alone and alway cross reference with sources I trust |
|  | It is useful for getting ideas and generating prose. Currently it doesn't seem accurate enough to make decisions and that still remains the responsibility of the clinician |
|  | I think currently patients will mostly still want to talk to a human rather than be diagnosed by AI. I do think there is significant upside to having an AI digital scribe to reduce time writing consultation notes and referrals. |
|  | No other comments, not rough experience on generative AI |
|  | unsafe, needs checking, mostly correct = difficult to spot errors |
|  | helpful so far - just been using a few days |
|  | I really know very little about it |
|  | Depends on budget to be rolled out |
|  | I am not aware of any of my patients using AI before thei appoinment. |
|  | Expanding technology in primary care |
|  | This is an exciting area but the ethics and legal aspects remain obscure |
|  | Been useful |
|  | i use generative a.i as a musician |
