# Supplementary material for: Generative Artificial Intelligence in Primary Care: Qualitative Study of UK General Practitioners’ Views
Source: J Med Internet Res. 2025 Aug 6;27:e74428. doi: 10.2196/74428 (PMC12327960; doi:10.2196/74428)
Supplement: Checklist 1 [file jmir-v27-e74428-s005.docx]

**Appendix** **2.** Checklist for Reporting Results of Internet E-Surveys (CHERRIES)

| ***Item Category*** | ***Checklist item*** | ***Described in the manuscript*** | ***Cited from the manuscript*** |
| --- | --- | --- | --- |
| **Design** | Describe survey design | Yes | In **Methods** section |
| **IRB (Institutional Review Board) approval and informed consent process** | IRB approval | Yes | **Methods > Ethical considerations**  The study received ethical approval from the Faculty of Psychology, University of Basel, Switzerland (approval ID: #030-24-1). |
|  | Informed consent | Yes | **Methods > Ethical considerations**  Participants were assured of anonymity, and informed consent was obtained before participation. |
|  | Data protection | Yes | **Methods > Ethical considerations**  The survey was hosted on Doctors.net.uk's secure platform, and all responses were encrypted and anonymized before data analysis. Email addresses and personal identifiers were removed prior to data transfer to the research team. The study complied with the European Union’s General Data Protection Regulation (GDPR). |
| **Development and pre-testing** | Development and testing | Yes | **Methods > Main survey** Prior to launch, the survey underwent pretesting and a pilot phase involving five UK-based GPs to assess usability and clarity. |
| **Recruitment process and description of the sample having access to the questionnaire** | Open survey versus closed survey | Yes | **Methods > Main survey**  In summary: we conducted an online survey among a random general practitioners (GPs) registered with Doctors.net.uk, the largest professional network for UK doctors affiliated with the General Medical Council (GMC). |
|  | Contact mode | Yes | **Methods** > Main survey  Invitations to a random sample of GPs who are members of Doctors.net.uk were distributed via email notifications or homepage advertisements on Doctors.net.uk, depending on user preferences. |
|  | Advertising the survey | Yes | **Methods > Main survey**  The survey was part of a recurring monthly omnibus survey, which maintains a fixed sample size of 1,000 participants. |
| **Survey administration** | Web/E-mail | Both | **Methods > Main survey**  Invitations to a random sample of GPs who are members of Doctors.net.uk were distributed via email notifications or homepage advertisements on Doctors.net.uk, depending on user preferences. |
|  | Context | Yes | **Methods > Main survey**  At the time of the study, Doctors.net.uk had 254,741 members, representing approximately 65% of the 390,000 registered doctors in the UK. |
|  | Mandatory/voluntary | Voluntary | **Methods > Ethical considerations** Participants were assured of anonymity, and informed consent was obtained before participation. |
|  | Incentives | Yes | **Methods > Ethical considerations** As an incentive, participants received a £7.50 (US$8.80, €8.83) shopping voucher upon survey completion. |
|  | Time/Date | Yes | **Methods > Main survey** The survey was open from January 7, 2025, to January 26, 2025. |
|  | Randomization of items or questionnaires | No |  |
|  | Adaptive questioning | No |  |
|  | Number of Items | No |  |
|  | Number of screens (pages) | No |  |
|  | Completeness check | Yes | **Methods > Ethical considerations** As an incentive, participants received a £7.50 (US$8.80, €8.83) shopping voucher upon survey completion. |
|  | Review step | No |  |
| **Response rates** | Unique site visitor (Unique site visitor = number of unique visitors to the survey itself) | Yes | **Results** A total of 1,141 unique visitors accessed the first page of the survey, and this number was used as the reference point for calculating view and participation rates |
|  | View rate (Ratio of unique survey visitors/unique site visitors) | Yes | **Results** The view rate was considered 100% (1,141/1,141 x 100), reflecting direct exposure from both email and website recruitment channels |
|  | Participation rate (Ratio of unique visitors who agreed to participate/unique first survey page visitors) | Yes | **Results** Of these, 1,067 respondents provided consent to participate by selecting “Yes” to the initial consent question, resulting in a participation rate of 94% (1,067/1,141 x 100). |
|  | Completion rate (Ratio of users who finished the survey/users who agreed to participate) | Yes | **Results** Among those who consented, 1,005 respondents completed the full survey, yielding a completion rate of 94% (1,005/1,067 x 100). |
| **Preventing multiple entries from the same individual** | Cookies used | No | Not used as a means to prevent multiple entries. |
|  | IP check | No | Not used as a means to prevent multiple entries. |
|  | Log file analysis | No | Not used as a means to prevent multiple entries. |
|  | Registration | Yes | **Methods > Main survey**  Registration was used to prevent multiple entries and to ensure access to only those potential respondents meeting the survey requirements. |
| **Analysis** | Handling of incomplete questionnaires | Yes | **Methods > Main survey** Respondents were required to complete all closed-ended questions to submit their responses; however, response to a single open-ended, free text question was optional. |
|  | Questionnaires submitted with an atypical timestamp | No |  |
|  | Statistical correction | No |  |

Reference:

Eysenbach G. Improving the quality of Web surveys: the Checklist for Reporting Results of Internet E-Surveys (CHERRIES). J Med Internet Res 2004;6:e34.
